# Supplementary figures and images for: Ac2-26 attenuates hepatic ischemia-reperfusion injury in mice via regulating IL-22/IL-22R1/STAT3 signaling
Source: PeerJ. 2022 Sep 28;10:e14086. doi: 10.7717/peerj.14086 (PMC9526407; doi:10.7717/peerj.14086)

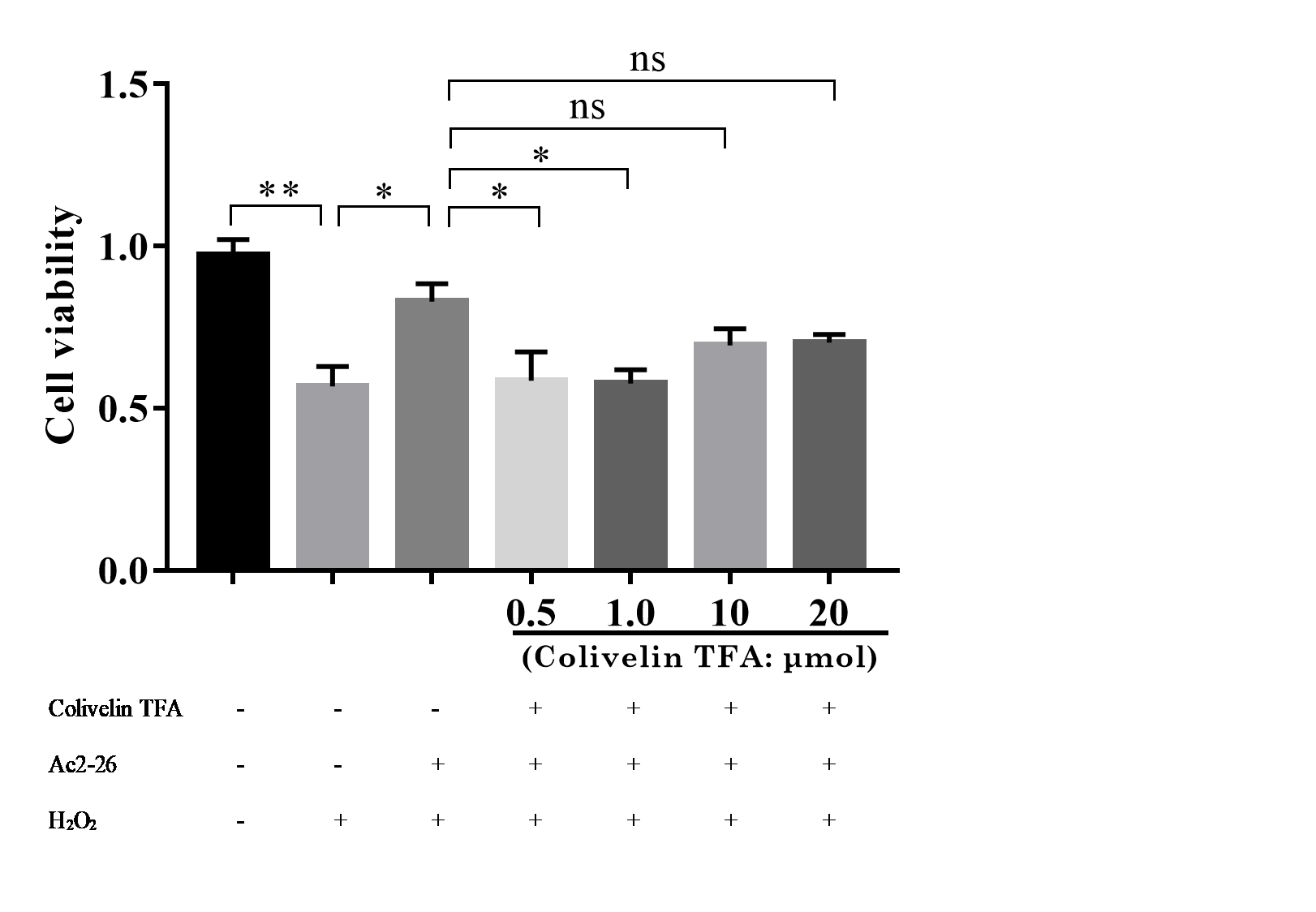

Supplement: Supplemental Information 4 [file peerj-10-14086-s004.zip › Figure S1, S2, and S3 raw data/Figure S1.png]

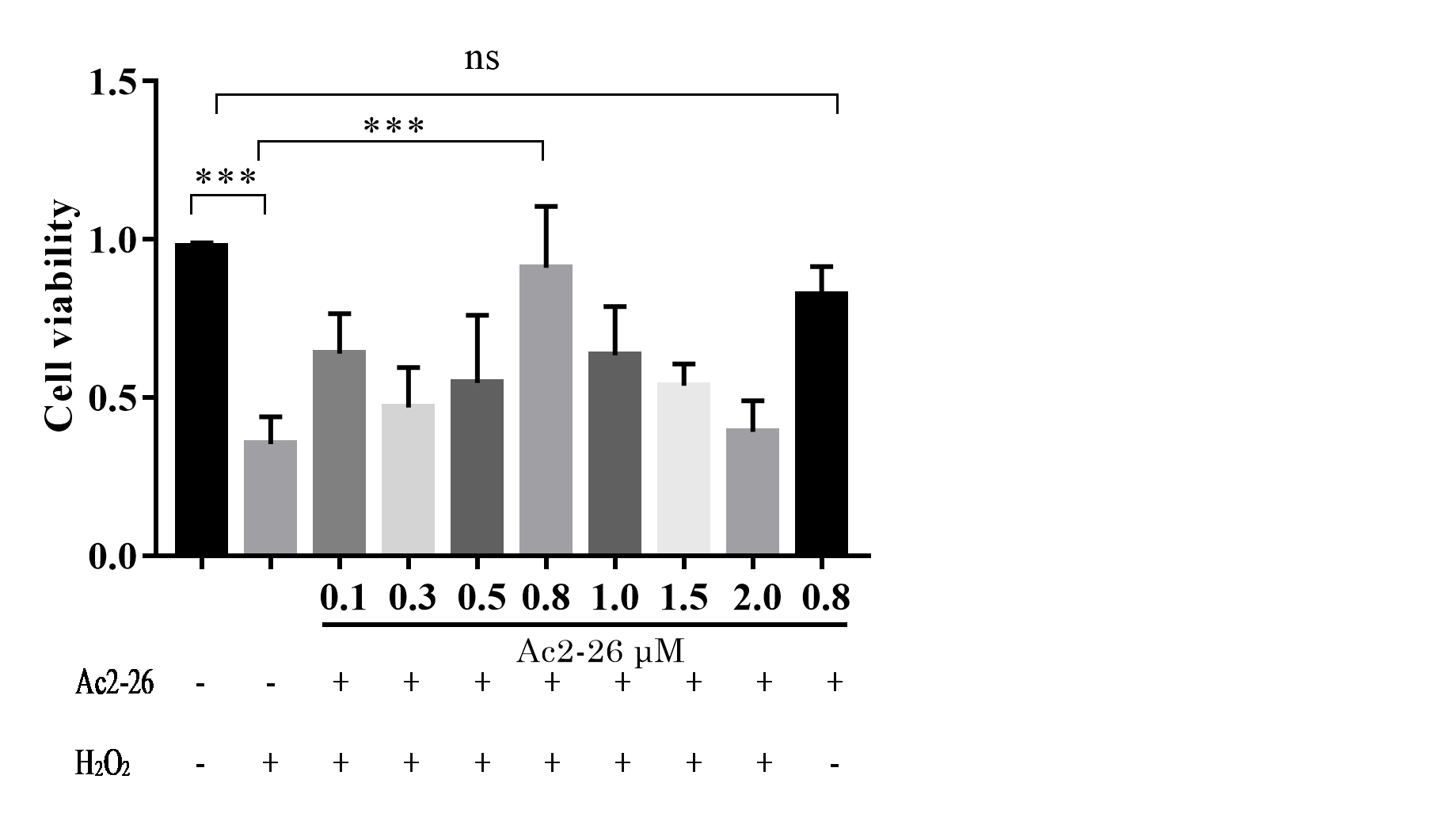

Supplement: Supplemental Information 4 [file peerj-10-14086-s004.zip › Figure S1, S2, and S3 raw data/Figure S2.png]

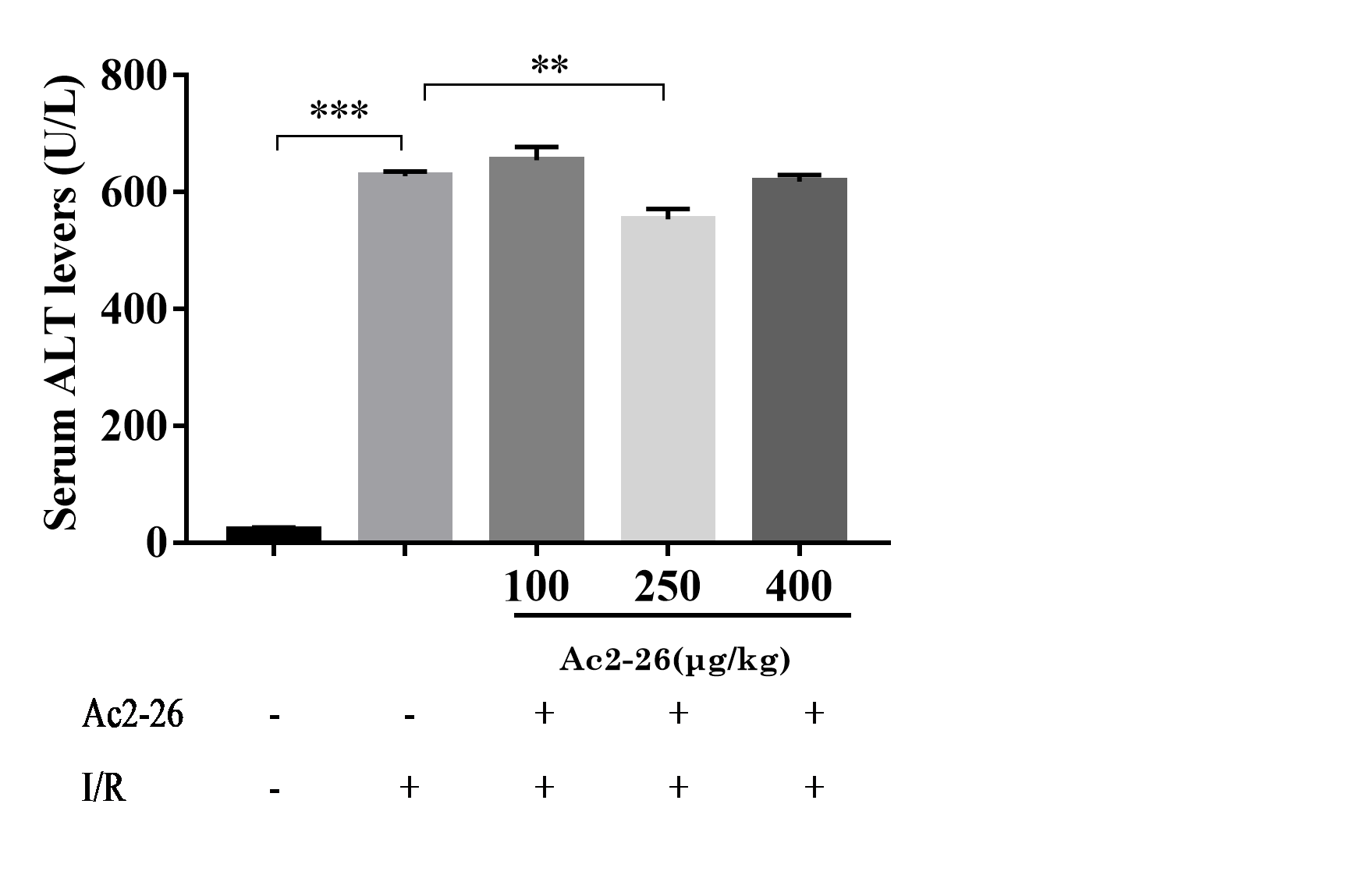

Supplement: Supplemental Information 4 [file peerj-10-14086-s004.zip › Figure S1, S2, and S3 raw data/Figure S3.png]

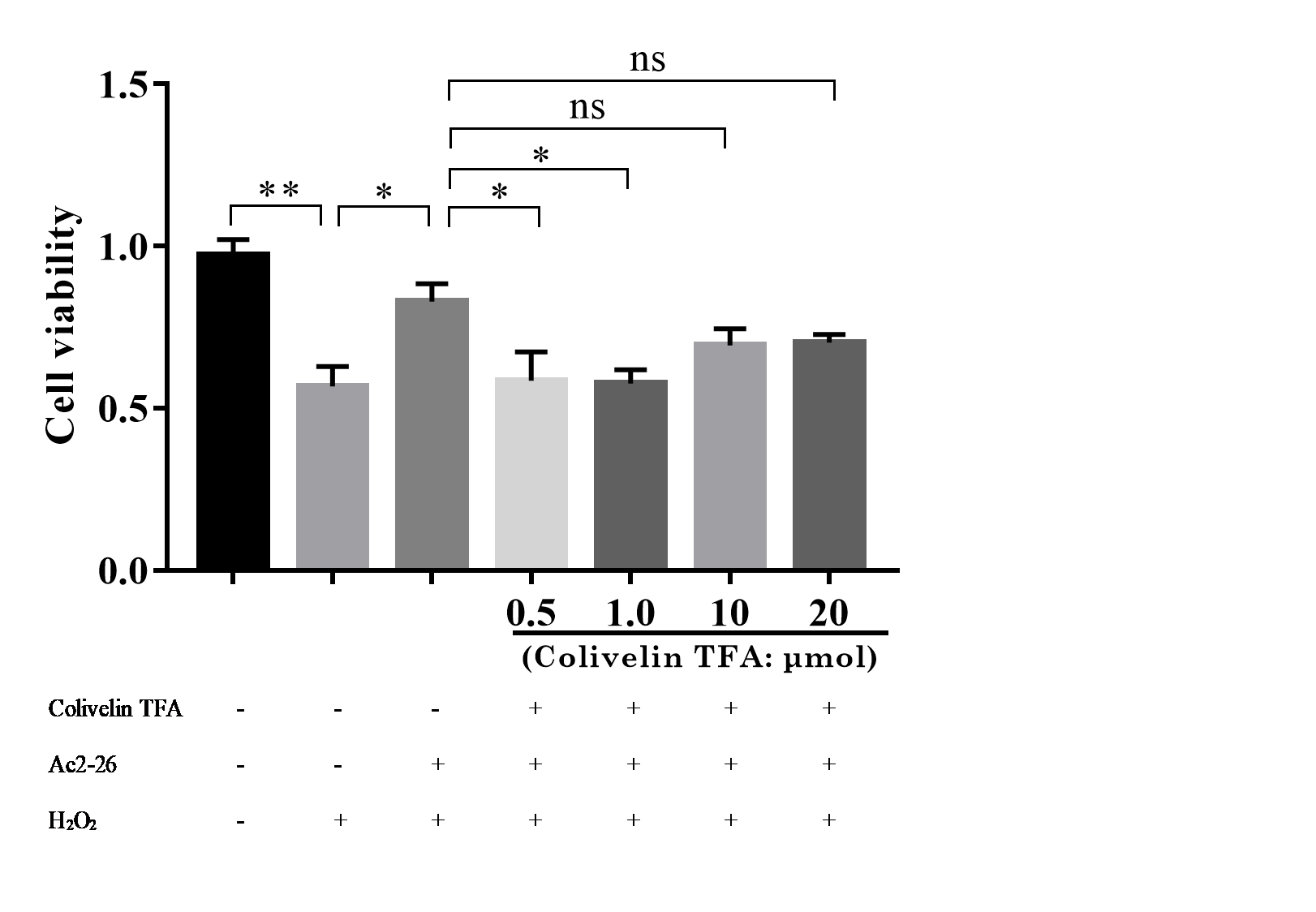

Supplement: Supplemental Information 5 [file peerj-10-14086-s005.png]

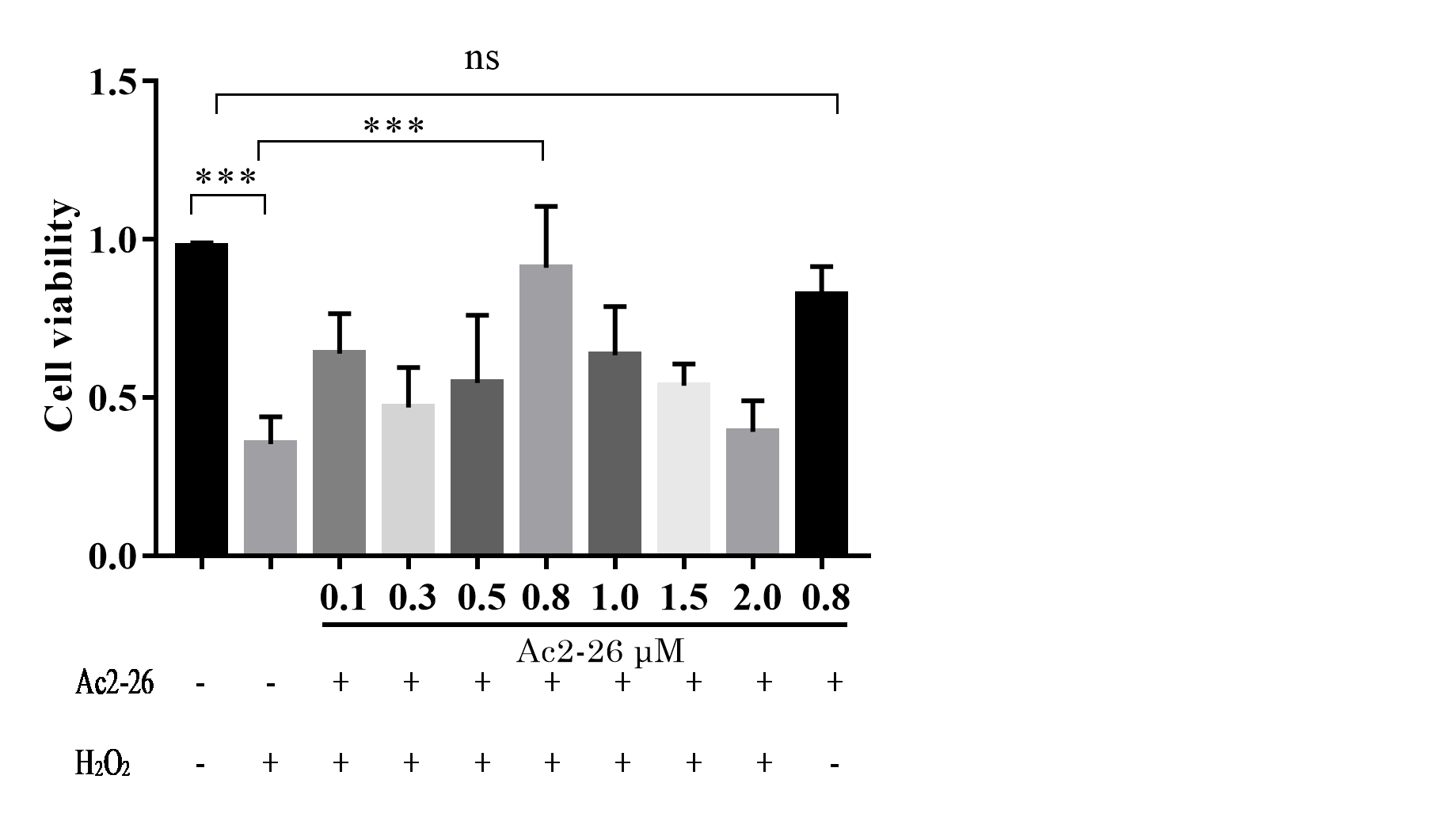

Supplement: Supplemental Information 6 [file peerj-10-14086-s006.png]

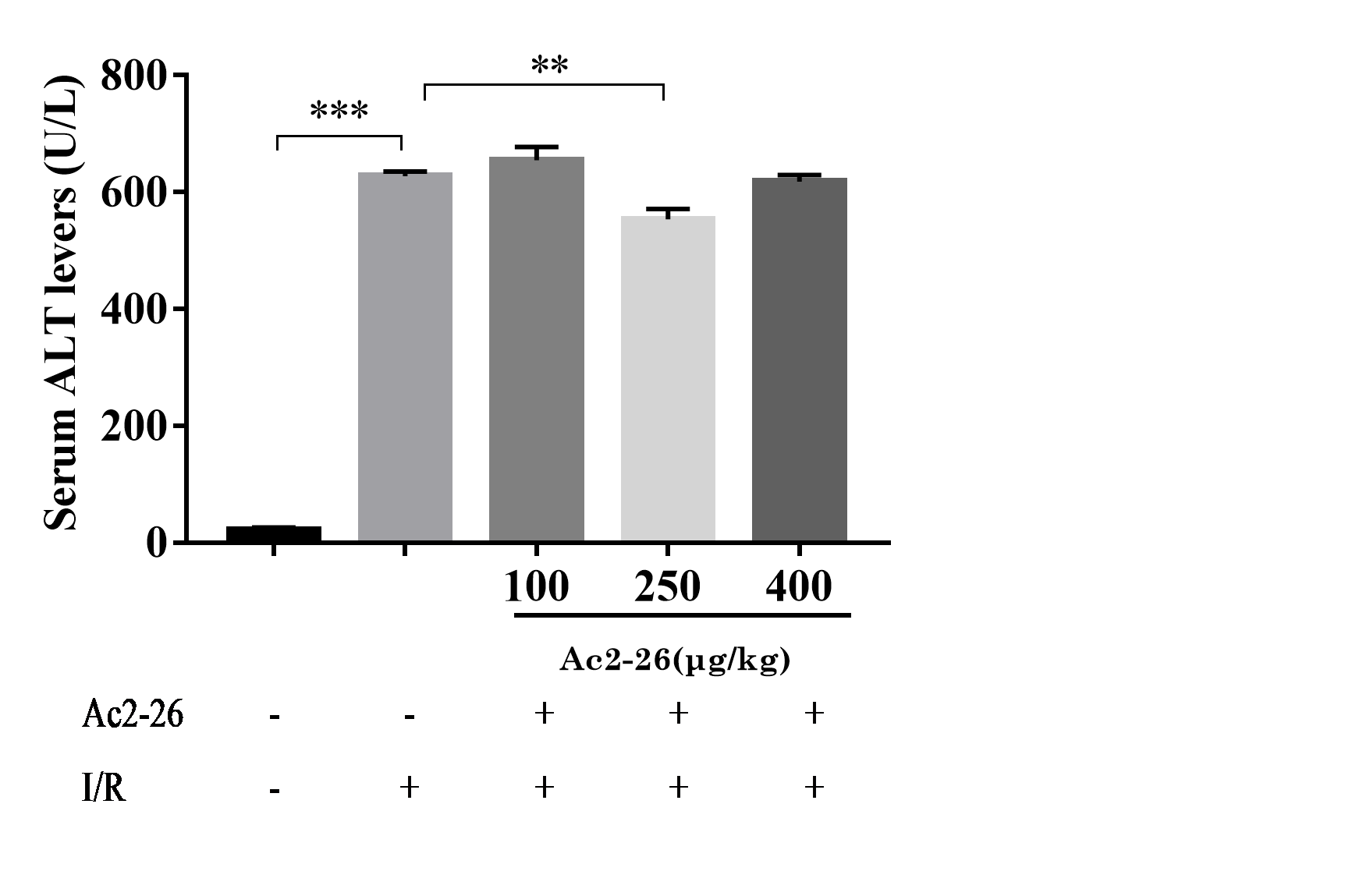

Supplement: Supplemental Information 7 [file peerj-10-14086-s007.png]
